# Generating Job Recommendations for People With Schizophrenia Spectrum Disorder Using Gemini 2.0 Flash and Claude Sonnet 4: An Exploratory Analysis

**DOI:** 10.1049/htl2.70058

**Published:** 2026-02-11

**Authors:** Maximin Lange, Nikolaos Koutsouleris, Ben Carter, Ricardo Twumasi

**Affiliations:** ^1^ Institute of Psychiatry, Psychology & Neuroscience King's College London London UK; ^2^ Department of Psychiatry and Psychotherapy Ludwig Maximilian University Munich Germany; ^3^ Max Planck Fellow Group Precision Psychiatry Max Planck Institute of Psychiatry Munich Germany

**Keywords:** patient rehabilitation, patient treatment, personnel, web services

## Abstract

Employment is a crucial part of recovery for individuals with severe mental illness. Individual placement and support (IPS) is the gold standard for vocational rehabilitation, yet IPS reaches only a fraction of who could benefit. Large language models (LLMs) have been proposed as potential tools for vocational guidance, but their utility for vulnerable populations is unknown. We conducted an analysis of LLM‐generated job recommendations for individuals with schizophrenia spectrum disorders, and for a matched control cohort without psychiatric diagnoses. We used discharge summaries from 450 patients with a primary diagnosis of schizophrenia spectrum disorder and 50 control cases in the MIMIC‐IV database, fitting three independent job recommendations per case with Gemini 2.0 Flash and Claude Sonnet 4. Recommendations were summarised as a frequency and LLM‐automated content analysis was used to analyse reasoning patterns, workplace accommodations, and alignment with supported employment principles. Both, Gemini and Claude, showed little diversity and strong bias toward entry‐level roles. In the schizophrenia cohort, Gemini mostly recommended data entry and other clerical jobs while Claude produced a similarly narrow pattern with the majority suggesting library‐related. The controls revealed comparable clustering, with Gemini defaulting to clerical work and medical secretary roles, and Claude to customer service. There was limited diversity in the role settings, which almost uniformly suggested flexible schedules and minimal social interaction. Nor was there diversity in how roles were tailored to patient strengths, qualifications, or prior experience; instead, demographic stereotypes such as age‐based framing, gendered role allocation, and assumptions about language skills often shaped the recommendations. Based on our data and procedures, preliminary evidence does not support immediate deployment of LLMs for job recommendations for the tested population; further evaluation is needed after integrating human oversight and bias‐mitigation steps.

## Graphical abstract

This study explores the use of large language models (Gemini 2.0 Flash and Claude Sonnet 4) to generate job recommendations for individuals with schizophrenia spectrum disorders using de‐identified clinical data from MIMIC‐IV. Both models produced narrow, stereotyped, and deficit‐oriented outputs, largely limited to entry‐level roles. These findings highlight risks of bias in applying AI to vocational planning and underscore the need for IPS‐aligned design before real‐world deployment.

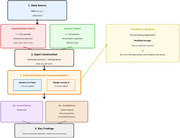

## 1 Background

### 1.1 Employment and Mental Health Benefits

Pharmacological treatments remain a cornerstone in treating severe mental illness such as schizophrenia [1, 2, 3]. Still, there is increasing evidence supporting the need for a more holistic approach to mental health care, addressing biological, psychological, and social factors [4, 5, 6, 7, 8, 9].

Competitive employment is continuously associated with reductions in psychiatric and physical symptoms, and improvements in self‐esteem, quality of life, and social functioning among individuals with severe mental illness [10, 11, 12, 13]. Employment offers a daily routine that can help stabilise mood and provides a sense of purpose and identity beyond a mental health diagnosis, fostering long‐term recovery [10, 11, 12, 13, 14, 15, 16, 17].

Whereas pharmacological treatment strategies for individuals with schizophrenia often rely on patients continuing prescriptions for antipsychotic medicine, sustained employment is associated with reduced reliance on mental health services and decreased healthcare costs over time [18].

Employment can hence be viewed as an effective and established health intervention for individuals experiencing mental illness, which achieves its benefits without negative side effects, which no other intervention, including pharmacological therapy, can deliver [19].

### 1.2 Individual Placement and Support

Over the past three decades, individual placement and support (IPS) has emerged as the most robust evidence‐based approach to helping people with severe mental illnesses achieve competitive employment [20, 21, 22, 23]. IPS targets competitive employment, meaning regular jobs that anyone can apply for, that pay at least minimum wage with the same compensation as workers without mental illness, performing the same duties, and that have no artificial time limits imposed by social service agencies [24, 25]. This includes employment across the full spectrum of occupations, from entry‐level positions to roles in high‐pressure environments, reflecting the principle that individuals with severe mental illness can succeed in mainstream competitive employment when provided appropriate support [26, 27].

The evidence base for IPS is strong. There are 27 randomised controlled trials across multiple countries consistently demonstrating IPS's superiority over other vocational interventions [28, 29]. Systematic reviews and meta‐analyses consistently show IPS enabling approximately 50%–65% of participants to obtain competitive employment, compared to 15%–25% in control groups receiving standard vocational services [23, 30, 31].

Importantly, these benefits extend beyond employment: participants in IPS programs experience fewer psychiatric hospitalisations, reduced healthcare costs, and improved clinical outcomes, with employment itself being associated with fewer mental health symptoms and enhanced quality of life [24, 32, 33, 34, 35, 36, 37]. IPS is widely regarded as the gold standard vocational rehabilitation model for individuals with severe mental illness, and its implementation is increasingly extending into broader disability populations [20, 37, 38, 39].

However, access to IPS remains limited. During 2022/23, in the UK, the National Health Service (NHS) reports the number of patients living with severe mental illness seen by IPS specialists to be around 8% of eligible patients, and only 5% for those suffering from substance disorder [40, 41]. Even where IPS programs exist, maintaining high fidelity to the model requires staff to receive ongoing technical assistance, training, and quality assurance, which are often unavailable in routine practice settings [21, 22].

### 1.3 The Role of Large Language Models

In recent years, advancements in Large Language Models (LLMs), have opened new avenues for innovation in mental healthcare [42, 43, 44]. These models have demonstrated sufficient capabilities in understanding and generating human‐like text, processing vast amounts of information, and performing summarisation, information retrieval, and even acting as conversational assistants in therapeutic contexts [44, 45, 46].

Building on these capabilities, LLMs can be used to generate personalised job recommendations [47, 48].

Applying such technology to interpret clinical notes for vocational insights represents a novel, though underexplored, extension of this concept. This investigation is, to our knowledge, the first systematic evaluation of off‐the‐shelf large language models generating job recommendations for individuals with schizophrenia spectrum disorders using real‐world clinical text.

### 1.4 Study Rationale

Given the substantial access gap for IPS, there is a need to explore alternative or complementary approaches that could provide vocational guidance to a population currently without access to evidence‐based services. This creates two scenarios that warrant investigation:

**Clinicians without access to IPS programs**: Psychiatrists and other mental health professionals might use LLMs to provide initial job recommendations based on their patients' clinical histories. This could offer a practical first step in employment‐focused recovery planning, particularly in settings where IPS services are unavailable or have long waiting lists.
**Patients seeking employment guidance independently**: Given the accessibility and popularity of LLMs, many individuals with schizophrenia spectrum disorders may already be turning to these tools for career guidance on their own.

The quality and appropriateness of such tools for this population remains unknown. Understanding the nature and quality of LLM‐generated job recommendations requires investigation for patient safety.

Given the documented risks of bias and the preliminary nature of such applications [49, 50, 51], this study adopts an exploratory approach. This initial exploration aims for understanding the potential and pitfalls before any consideration of future validation or real‐world application.

## 2 Methods

### 2.1 Job Recommendation Generation

#### 2.1.1 Patients

We used MIMIC‐IV [54] v. 3.1, a publicly available database containing deidentified electronic health records from over 360,000 individual patients admitted to the Beth Israel Deaconess Medical Center (BIDMC) in Boston, MA, USA, between 2008 and 2022.

##### 2.1.1.1 Experimental Group

For eligibility, patients had to meet the following criteria: aged 18–40 years (in accordance with previous works [53, 54]); Primary ICD 9 or 10 diagnosis of schizophrenic, schizophreniform, or schizoaffective disorder (ICD9 295.X or ICD10 F2X) and having a discharge summary available in the database. SQL code for extraction can be found at github.com/maximinl/gemini‐job‐reco.

##### 2.1.1.2 Control Group

For the control group, patients had to meet the following criteria: aged 18–40 years; primary ICD‐9 or ICD‐10 diagnosis not related to psychiatric, behavioural, or neurocognitive disorders (all ICD‐10 F codes and ICD‐9 codes 290–319 were excluded); and having a discharge summary available in the database. Only the first admission per patient, and the corresponding primary diagnosis was retained. To construct a reproducible sample, we applied deterministic randomisation with a fixed seed and selected 50 unique patients. SQL code for extraction can be found at github.com/maximinl/gemini‐job‐reco.

#### 2.1.2 Software

##### 2.1.2.1 LLM and API Configuration

Job recommendations were generated using the Gemini 2.0 Flash model (identifier: models/gemini‐2.0‐flash) via the Google Generative AI API for Python (google‐generativeai library). Model generation parameters were configured as: temperature = 0.6, top_p = 1.0, top_k = 40, and maximum output tokens = 1536. Standard safety settings were applied to block harmful content (categories: Harassment, Hate Speech, Sexually Explicit, Dangerous Content; threshold: BLOCK_MEDIUM_AND_ABOVE).

For comparison, job recommendations were also generated using the Claude Sonnet 4 model (identifier: claude‐sonnet‐4‐20250514) via the Anthropic API (anthropic library). Model generation parameters were configured as: temperature = 0.6, maximum output tokens = 1536, with default safety filters enabled to block harmful or unsafe outputs (covering harassment, hate speech, sexually explicit, and dangerous content). Unlike Gemini, Claude does not support top_p or top_k sampling parameters, and its sampling behaviour relies primarily on temperature and maximum output length.

We used Gemini and Claude since they are the only mainstream LLM that do not use prompts and responses as data to train its models, which makes it eligible for use of MIMIC IV data as per responsible use guidelines [57].

##### 2.1.2.2 Recommending Jobs

We follow previous works [58], in letting the LLM parse each patient multiple times. The following steps are repeated three times for each patient to explore response variability:

A prompt was dynamically constructed for each run, providing the model with the patient's discharge summary and demographic information (age, sex, ethnicity). The prompt instructed the model to act as a career counsellor and perform three tasks:

1. Recommend one specific job role suitable for potential workforce re‐entry or accommodation needs, explicitly avoiding generic program names (e.g., ‘vocational rehabilitation’) for this specific output.
2. Explain the reasoning, referencing the discharge summary and demographics.
3. Mention potential challenges or considerations related to the patient's background for the recommended role.

The constructed prompt was sent to the API. The full text response generated by the model for each successful run, along with demographics, run ID, timestamp, model name, and generation configuration, was saved incrementally. To see the prompt formulation verbatim, please visit github.com/maximinl/gemini‐job‐reco.

##### 2.1.2.3 Prompt‐Tuning Sensitivity Test

To examine whether clustering could be mitigated through lightweight prompt adjustments, all schizophrenia‐condition cases were re‐run using Gemini 2.0 Flash with an additional instruction to “vary the job type and industry across different cases.”

**FIGURE 1:**
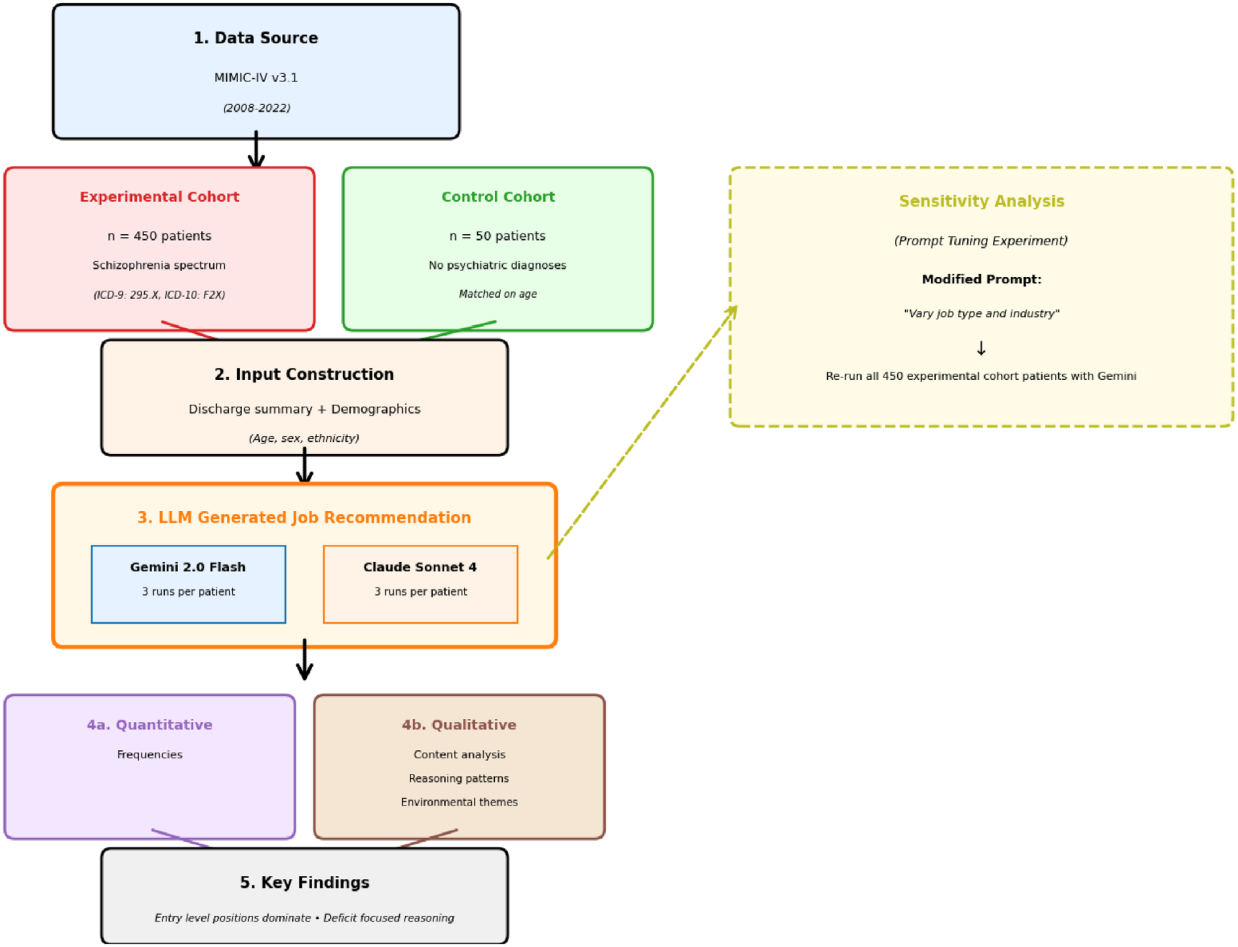
Schematic experimental workflow.

Full experimental workflow can be seen in Figure 1.

### 2.2 Job Recommendation Evaluation

#### 2.2.1 Quantitatively

Each of the recommendations were summarised as frequency.

#### 2.2.2 Content Analysis

We employed a fully automated qualitative content analysis approach using both Gemini and Claude for their corresponding answers. This follows recent methodological advances in LLM‐assisted qualitative research [57, 58, 59, 60, 61].

Large language models have been reported to achieve human‐level performance in various text analysis tasks, for example, outperforming crowd workers and matches trained experts in text annotation tasks [62, 63], surpassing both experts and crowd workers in political text classification, and in qualitative coding specifically, achieved human‐equivalent performance across multiple coding tasks [66] and more than 90% agreement between LLM and human coders [61]. These findings suggest that LLMs have reached a level of sophistication where they can reliably perform complex interpretive tasks traditionally reserved for human analysts

Our analysis utilised a single‐prompt synthesis approach where the LLM was tasked with performing content analysis across five predefined analytical categories: (1) job role recommendations, (2) work environment characteristics, (3) justifications, (4) challenges/accommodations, and (5) consistency assessment.

Unlike traditional content analysis that requires human coders to iteratively develop and apply coding schemes, our approach leveraged the LLM's capability to simultaneously identify, categorise, and synthesise patterns across multiple responses in a single analytical pass [61, 64]. The LLM was instructed to focus on patterns, consensus, and variations rather than simple enumeration, incorporating elements of thematic synthesis within a content analytical framework. This fully automated approach represents autonomous qualitative data analysis, where the entire analytical process, from initial categorisation through synthesis, is performed by the LLM [62]. While this methodology trades the iterative refinement and contextual depth of traditional qualitative analysis for scalability and efficiency, it enables rapid synthesis of complex qualitative data that would be impractical for manual analysis.

### 2.3 (Initially Planned): Expert Validation

While this study provides initial insights into LLM‐generated job recommendations for individuals with schizophrenia, comprehensive validation would require systematic evaluation by qualified IPS specialists and workplace accommodation experts. The goal of such research should be to inform the development of AI‐assisted tools that could complement rather than replace evidence‐based supported employment services, potentially expanding access to vocational guidance while maintaining appropriate clinical oversight and integration with comprehensive mental health care.

However, this validation approach might be, for now, superfluous with current models, given the findings of our analysis. This will be discussed in detail in the results and discussion sections.

## 3 Results

### 3.1 Patients Included

#### 3.1.1 Experimental Group

We screened 364,627 unique patients in MIMIC IV for study eligibility between 2008 and 2022; 450 patients fulfilled inclusion criteria and were extracted from the database.

Most patients were male (68.9%) and of White ethnicity (40%; Table 1a).

**TABLE 1a:** Experimental cohort.

Characteristic		N
Total patients		450
Age (SD)	28.3 ± 6.3	450
Gender		
M	68.9%	310
F	31.1%	140
Ethnicity		
White	40%	180
Black/Afr. Amer.	35.1%	158
Hispanic/Latino	8.2%	37
Asian	8.7%	39
Other	5.8%	26
Unknown	2.2%	10

**TABLE 1b:** Control cohort.

Characteristic		N
Total patients		50
Age (SD)	29.0 ± 6.5	50
Gender		
M	36.0%	18
F	64.0%	32
Ethnicity		
White	50.0%	25
Black/Afr. Amer.	30.0%	15
Hispanic/Latino	8.0%	4
Asian	2.0%	1
Other	4.0%	2
Unknown	6.0%	3
Primary diagnosis
Obstetric/gynaecological	24.0%	12
Trauma/injury	14.0%	7
Gastrointestinal	20.0%	10
Infectious diseases	14.0%	7
Cardiovascular	6.0%	3
Musculoskeletal	6.0%	3
Neurological	8.0%	4
Dermatologic	4.0%	2
Hematologic/immunologic	2.0%	1
Adverse reactions/complications	2.0%	1

#### 3.1.2 Control Group

Patients were similar in age (29.0 ± 6.5) to the experimental group. Most were female (64%), Half of the sample were White (50.0%), with further representation from Black/African American (30.0%), Primary discharge diagnoses reflected a broad distribution across medical domains. The most frequent were obstetric/gynaecological conditions (24.0%), gastrointestinal disorders (20.0%), trauma or injury (14.0%), and infectious diseases (14.0%) Table 1b.

### 3.2 Job Recommendations

#### 3.2.1 Gemini

##### 3.2.1.1 Experimental Group

###### 3.2.1.1.1 Frequencies

For each of the 450 patients, three independent recommendations were generated, yielding 1350 total outputs. A total of 112 distinct job titles were recommended. However, the distribution was highly skewed. The single most frequent recommendation was data entry clerk (499 instances; 37.0%), followed by library assistant (237; 17.6%) and peer support specialist (68; 5.0%; Table 2).

**TABLE 2:** Job recommendations Gemini.

Rank	Job title	Frequency	Percentage
1	Data entry clerk	499	37.00%
2	Library assistant	237	17.60%
3	Peer support specialist	68	5.00%
4	Janitorial assistant	48	3.60%
5	Delivery driver	33	2.40%
6	Document shredding assistant	32	2.40%
7	Night security guard	30	2.20%
8	Landscaping assistant	21	1.60%
9	Remote data entry clerk	20	1.50%
10	Stock clerk	19	1.40%
11+	Others (102 different job types)	343	25.40%
Total	All job recommendations	1350	100.00%

###### 3.2.1.1.2 Qualitative Analysis

Overall, recommendations clustered around low‐complexity clerical and service positions, with limited representation of higher‐skill or higher‐prestige occupations.

The environmental considerations accompanying job recommendations were uniform. Across job types, recommendations consistently emphasized structured and predictable environments with clearly defined routines, quiet workspaces with minimal noise, limited social interaction, and flexibility in scheduling to accommodate medication adherence and fluctuations in symptoms.

Reasoning behind recommendations was predominantly deficit‐focused. Jobs were justified based on reducing stressors, minimizing social contact, or accommodating difficulties with concentration and symptom management. Clinical features such as hallucinations, paranoia, or disorganised thought were routinely linked to recommendations for simple, repetitive tasks. Strengths were less frequently emphasised. When mentioned, they tended to be limited to basic skills such as attention to detail, ability to follow routines, or having previously attended school. More personal strengths, interests, or ambitions were rarely incorporated. This resulted in reasoning that foregrounded limitations rather than adopting a recovery‐oriented perspective that might emphasize growth, self‐determination, or personal goals.

Commonly identified challenges included difficulties with maintaining focus, managing stress and anxiety, coping with interpersonal interactions, adhering to medication, and maintaining consistent attendance. Proposed supports were largely generic and standardised. Most focused on structuring tasks clearly, providing quiet workspaces, ensuring flexible schedules, or relying on supportive supervisors. More individualised or proactive supports, such as job coaching, skills training, or specific coping strategies, were rarely suggested. Strategies for addressing medication adherence or symptom management were similarly limited, often reduced to reminders or avoidance of potential triggers.

Despite substantial variation across patients in demographics and clinical histories, recommendations were often similar. Patients with different presentations were frequently offered identical jobs, particularly data entry and library assistant.

##### 3.2.1.2 Control Group

###### 3.2.1.2.1 Frequencies

We analysed job recommendations for a control cohort of 50 patients aged ≤40 years without psychiatric or neurocognitive diagnoses. A total of 150 recommendations were generated (3 per patient).

The distribution of recommendations was again highly skewed. Nearly half of all outputs were for Medical Secretary (71 of 150, 47.3%), followed by Remote Customer Service Representative (25 of 150, 16.7%). A second cluster of roles included Medical Scribe (4.0%), Medical Records Clerk (4.0%), and Medical Transcriptionist (4.0%; Table 3).

**TABLE 3:** Job recommendations Gemini control.

Rank	Job title	Frequency	Percentage
1	Medical secretary	71	47.30%
2	Remote customer service representative	25	16.70%
3	Medical scribe	6	4.00%
4	Medical records clerk	6	4.00%
5	Medical transcriptionist	6	4.00%
6	Medical records technician	5	3.30%
7	Data entry clerk	5	3.30%
8	Remote medical transcriptionist	3	2.00%
9	Technical writer	3	2.00%
10	Delivery driver	3	2.00%
11+	Others (11 different job types)	17	11.30%
Total	All job recommendations	150	100.00%

###### 3.2.1.2.2 Qualitative Analysis

Environmental recommendations emphasised supportive office environments for clerical roles, flexible scheduling (present in ∼70% of cases), ergonomic accommodations (∼60%), and quiet home‐based work for remote roles (∼30%). Access to restroom facilities (10%) and supportive supervisors (∼25%) were noted in specific contexts, particularly for patients with gastrointestinal issues or recent surgery.

Patients were often delegated to sedentary clerical roles, with strengths framed around “familiarity with healthcare” due to prior treatment. Communication skills, bilingual ability, and age (assumed adaptability in younger patients) were also invoked. However, this appeared to reflect stereotyped assumptions, such as recommending medical secretary disproportionately for women with obstetric/gynaecologic history.

Challenges identified included fatigue, pain, mobility limitations, and stress management. Few recommendations explicitly connected to long‐term recovery, focusing instead on accommodations for symptom management.

#### 3.2.2 Claude

##### 3.2.2.1 Experimental Group

###### 3.2.2.1.1 Frequencies

For each of the 450 patients, three independent recommendations were generated, yielding 1350 total outputs. A total of 148 distinct job titles were recommended. The distribution was again highly skewed. Nearly half of all recommendations were library‐related roles, with library assistant—technical services (293; 21.7%) and library page/shelving assistant (184; 13.6%) dominating (Table 4).

**TABLE 4:** Job recommendations Claude.

Rank	Job title	Frequency	Percentage
1	Library assistant—technical services	293	21.70%
2	Peer support specialist	190	14.10%
3	Library page/shelving assistant	184	13.60%
4	Library assistant	79	5.90%
5	Library assistant—shelving and materials processing	46	3.40%
6	Warehouse associate—inventory support	40	3.00%
7	Peer support specialist in mental health services	36	2.70%
8	Library shelving assistant	29	2.10%
9	Warehouse package handler	28	2.10%
10	Research data entry specialist	20	1.50%
11+	Others (138 different job types)	405	30.00%
Total	All job recommendations	1350	100.00%

###### 3.2.2.1.2 Qualitative Analysis

Overall, recommendations clustered around a small set of low‐complexity roles, especially library and peer support work. Higher‐skill, professional, or supervisory roles were virtually absent. Environmental requirements were remarkably consistent across job types, with most recommendations emphasising quiet and structured workplaces, flexible scheduling, supportive supervisors, minimal social interaction, and predictable routines. These appeared in over three‐quarters of sampled recommendations, suggesting a highly standardised template.

Reasoning behind job matching drew heavily on symptom‐based assumptions. Patients reporting hallucinations were directed to quiet library roles and those with concentration issues to structured routine tasks. Age also influenced framing: younger patients were described as having “growth potential,” whereas older patients were described in terms of “life experience.”

Strengths were acknowledged but often underutilised. Bilingual skills, prior professional experience, or higher education (including cases with legal training) were rarely incorporated into the actual job match. Instead, the logic emphasised limitations over recovery‐oriented growth.

Commonly cited challenges included medication adherence, concentration difficulties, and managing interpersonal interactions. Supports were largely generic: flexible schedules, regular supervisor check‐ins, written instructions, and gradual hour increases. While nearly 80% mentioned medication adherence, concrete workplace‐based strategies were rare.

##### 3.2.2.2 Control Group

###### 3.2.2.2.1 Frequencies

We analysed job recommendations for a control cohort of 50 patients aged ≤40 years without psychiatric or neurocognitive diagnoses. A total of 150 recommendations were generated (3 per patient).

Recommendations were concentrated in customer service roles. Within this category, remote and hybrid customer service representative positions predominated, alongside call‐centre and customer success variants (Table 5). This clustering mirrors the patterns observed in the schizophrenia cohort, with limited occupational diversity and a narrow emphasis on entry‐level positions.

**TABLE 5:** Job recommendations Claude Control.

Rank	Job title	Frequency	Percentage
1	Remote customer service representative	41	27.30%
2	Customer service representative—remote/hybrid	21	14.00%
3	Customer success coordinator	9	6.00%
4	Customer service representative—remote/call center	8	5.30%
5	Remote customer success specialist	8	5.30%
6	Remote customer success coordinator	7	4.70%
7	Customer service representative—remote/flexible schedule	5	3.30%
8	Remote technical support specialist	3	2.00%
9	Construction safety inspector	3	2.00%
10	Medical administrative coordinator	3	2.00%
11+	Others (25 different job types)	42	28.00%
Total	All job recommendations	150	100.00%

###### 3.2.2.2.2 Qualitative Analysis

Recommendation rationales were primarily demographic‐ and condition‐driven. Age was used as the universal starting point: younger patients were described as “adaptable” or “digitally skilled,” while older patients were framed as having “accumulated experience.” Medical conditions (e.g., recovery from surgery, chronic illness, pregnancy) often influenced recommendation rationales, often prioritising symptom accommodation over career growth. Demographic assumptions seemed evident, including bilingual placement for Hispanic/Latino patients and gendered assumptions linking women to communication‐heavy roles.

Commonly identified challenges included fatigue, appointment scheduling conflicts and concentration difficulties as well as stress sensitivity, and medication side effects. Support strategies focused on flexible scheduling, phased return‐to‐work plans, employee assistance programs, understanding supervisors, and health benefits.

Recommendations emphasised limitations in most cases. Demographic stereotyping was frequent, women were disproportionately steered into “nurturing” customer service roles, and older patients were framed as less adap. Recovery‐oriented language (e.g., personal growth, career advancement) was largely absent.

Accommodations and rationales repeated across cases. Personalisation was largely restricted to age bracket, acute medical condition, ethnicity, and gender, while patient interests, educational history, or occupational aspirations rarely influenced recommendations.

##### 3.2.2.3 Overall Summary

**FIGURE 2:**
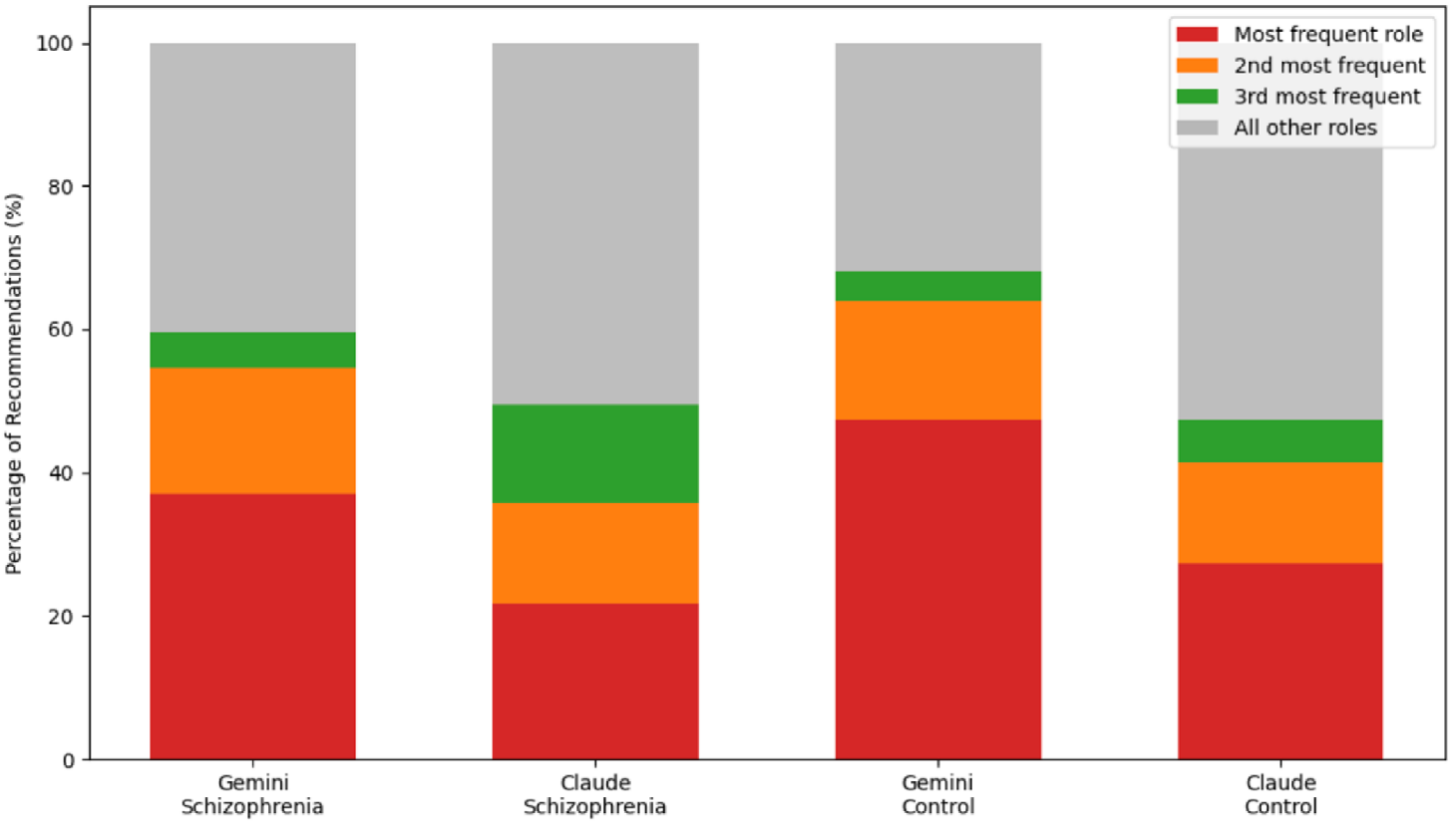
Occupational concentration by model and cohort.

Figure 2 provides a cross‐model visual comparison of recommendations’ occupational concentration. Across both LLMs and both cohorts, recommendations consistently converged on a narrow set of roles. This compression into a small number of job types occurred in both the schizophrenia and control groups.

**FIGURE 3:**
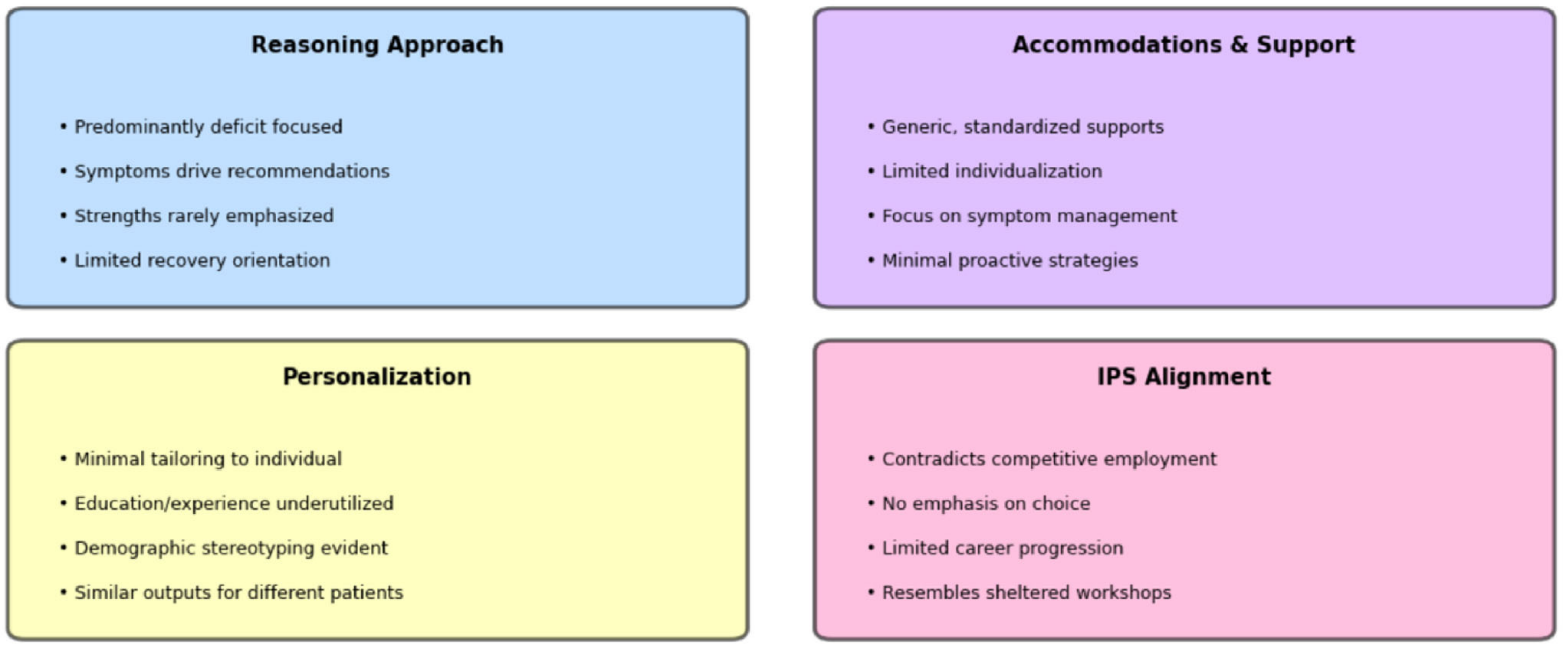
Summary of qualitative findings: Common patterns across all recommendations.

Figure 3 summarises the qualitative themes identified across models. Despite substantial variation in patient histories and demographics, the models produced highly uniform reasoning patterns, environmental recommendations, and accommodation templates. Both LLMs relied heavily on stereotyped assumptions, foregrounded symptoms and risks over strengths or aspirations, and made limited use of patients’ documented qualifications or prior work history. These common patterns complement the quantitative findings by showing that the narrow range of job titles is accompanied by similarly constrained explanatory logic and support structures.

##### 3.2.2.4 Prompt‐Tuning Sensitivity Test

Despite additional instruction to the models to “vary the job type and industry across different cases”, outputs remained highly repetitive (Table 6), and similar to the main execution.

**TABLE 6:** Sensitivity analysis: Prompt‐tuned Gemini results (schizophrenia cohort).

Rank	Job title	Frequency	Percentage (%)
1	Library assistant	644	47.70
2	Data entry clerk	88	6.52
3	Data entry clerk (remote)	63	4.67
4	Night stocker	60	4.44
5	Janitorial assistant	49	3.63
6	Landscaping assistant	32	2.37
7	Bilingual customer service representative (remote)	27	2.00
8	Peer support specialist	24	1.78
9	Peer support specialist (mental health)	23	1.70
10	Document shredding assistant	21	1.56
11 +	Others (104 different job types)	319	23.63
Total	All job recommendations	1350	100.00

## 4 Discussion

In this exploratory study, we examined the feasibility and characteristics of using Gemini 2.0 and Claude Sonnet 4 to generate job recommendations for individuals with schizophrenia spectrum disorders, based on real world clinical discharge summaries. Across a total of 1350 recommendations for 450 patients for each LLM, our analyses revealed preliminary evidence of algorithmic biases that contradict evidence‐based vocational rehabilitation principles.

Patients were repeatedly channelled toward routine, entry‐level jobs with limited variation. Both models produced similar environmental accommodations, typically quiet and structured workplaces, flexible scheduling, minimal social interaction, and supportive supervision. These accommodations appeared to be applied universally rather than tailored to individual needs.

Despite identical prompts, the two models diverged in analytic stance. Gemini's qualitative analysis remained largely descriptive, noting repetitive and deficit‐focused reasoning. Claude adopted a more explicitly critical lens, quantifying recurrence of environmental phrases, identifying age‐based framing patterns, and describing the outputs as a form of occupational segregation reminiscent of sheltered workshops. Claude also drew attention to the underutilisation of professional or educational histories, highlighting cases where highly trained patients were nonetheless directed toward entry‐level clerical tasks. Our prompt tuning sensitivity analysis further strengthens these conclusions. Despite explicit instruction to diversify recommendations, models appeared to substitute one stereotypical role for another while maintaining a narrow range of entry level positions. Addressing these biases would require more fundamental interventions, including fine tuning with carefully curated, bias mitigated datasets; integration of structured constraints aligned with IPS principles (e.g., requiring recommendations across multiple job complexity levels); or architectural modifications that explicitly separate medical information from occupational matching logic. To contextualise these findings, we conducted control experiments using a small subset of cases with no schizophrenia spectrum disorder, where a similar pattern emerged.

Our findings stand in tension with the established evidence that individuals with schizophrenia can succeed across the full spectrum of competitive employment when supported through Individual Placement and Support [24, 33, 65].

There are several plausible mechanisms that might explain our results. First, discharge summaries emphasise impairment, risk, and illness while rarely documenting strengths, interests, or vocational successes. Feeding such documents to a general‐purpose model encourages conservative and risk‐minimising recommendations. Second, the task design asked for exactly one job per case, funnelling the model toward the safest and most generic option rather than a balanced set of possibilities, or even the option of a reply such as ‘more information needed, this recommendation wouldn't be appropriate’. Third, the models’ pretraining and alignment may have amplified cultural stereotypes about mental illness and employability, further reinforced by reward structures that favour “safe” answers.

These findings extend the literature on algorithmic bias in healthcare [66, 67, 68] by demonstrating how disability‐related discrimination manifests in vocational recommendation by LLMs. The nature of the bias observed across both experimental and control cases suggests that current fairness‐oriented approaches to AI development may be insufficient for applications serving vulnerable populations.

There are several limitations to this study. We examined only two mainstream models and only patients from a single US hospital site, relying on discharge summaries that under‐document vocationally relevant strengths and aspirations. We did not analyse the training data or alignment procedures that may have contributed to the observed patterns. Moreover, Gemini‐generated recommendations were analysed by Gemini and Claude‐generated recommendations by Claude, introducing the possibility that one model's analytic lens influenced the interpretation of its own outputs.

Several design implications follow. Inputs should include structured information on strengths, interests, education, and explicit vocational goals to counterbalance the deficit orientation of discharge narratives. Task framing should move from producing a single job to generating a diverse shortlist across job families, with explicit inclusion of at least one aspirational role alongside pragmatic options. A second‐stage reranker, tuned to IPS‐aligned criteria such as choice, competitive employment, rapid search, and individualised supports, could down‐weight stereotyped or overly conservative outputs. Auditing should monitor distributions of outputs by job family and complexity, with safeguards against undue concentration. Human‐in‐the‐loop review by IPS specialists and people with lived experience should remain a non‐negotiable component before any practical use.

Future research should broaden the scope to hypothesis driven statistical analyses, comparing recommendations in more detail. Additional datasets, and evaluation frameworks, ideally involving IPS practitioners and service users directly are needed. Investigations should also examine whether prompt engineering, fine‐tuning with bias‐aware datasets, or alternative architectures can counteract systematic biases and generate more appropriate and recovery‐oriented recommendations.

In conclusion, both Gemini 2.0 Flash and Claude Sonnet 4 exhibited bias when generating vocational recommendations for individuals with schizophrenia spectrum disorders, and control analyses demonstrate that these tendencies generalise beyond psychiatric contexts. The consistent concentration of outputs into a narrow occupational sphere, coupled with templated environmental accommodations, suggests that current LLMs underestimate the capabilities of individuals with severe mental illness and more broadly perpetuate a systemic form of occupational segregation.

## Author Contributions

**Maximin Lange**: conceptualisation, methodology, software, data curation, validation, formal analysis, investigation, writing – original draft, visualisation, project administration. **Nikolaos Koutsouleris**: funding acquisition, writing – review & editing, supervision. **Ben Carter**: methodology, validation, formal analysis. **Ricardo Twumasi**: funding acquisition, supervision, writing – review & editing.

## Funding

The authors have nothing to report.

## Ethics Statement

This research was conducted in accordance with the Declaration of Helsinki and followed international ethical standards for medical research involving human data. MIMIC‐IV data was collected as part of routine clinical care. It has been deidentified and transformed. MIMIC data is approved for research by the institutional review boards of the Massachusetts Institute of Technology and Beth Israel Deaconess Medical Center, who granted a waiver of informed consent and approved the sharing of the research resource. It is available to researchers who have completed training in human research and signed a data use agreement.

## Conflicts of Interest

Maximin Lange has received an International Travel Grant from the IET. https://www.theiet.org/media/12cb44f4/international‐travel‐travel‐report‐from‐maximin‐lange‐may‐2025.pdf

## Data Availability

Source Code for data extraction and all analysis reported can be found at https://github.com/maximinl/gemini‐job‐reco In this study, we used MIMIC‐IV version 3.1 https://doi.org/10.13026/kpb9‐mt58. For more information on MIMIC, please visit https://mimic.mit.edu. For MIMIC IV data access, researchers must register with PhysioNet and get approval for dataset access after appropriate training: https://physionet.org.
